# HABS-BLOCKS© Inhibited *Microcystis* and *Planktothrix* and Reduced Microcystin Concentrations in a Lake Water Mesocosm Study

**DOI:** 10.3390/microorganisms13051074

**Published:** 2025-05-05

**Authors:** Cameron Gastaldo, Stephen Vesper

**Affiliations:** United States Environmental Protection Agency, Center for Environmental Measurement and Modeling, 26 West M. L. King Drive, Cincinnati, OH 45268, USA; gastaldo.cameron@epa.gov

**Keywords:** bloom, glucose, cyanobacteria, microcystin, nitrogen

## Abstract

Toxins produced by cyanobacteria are a threat to drinking water and the aquatic ecosystem. Previously, we reported that adding glucose to lake water could reduce cyanobacterial populations. To target the glucose to the euphotic zone, floating HABS-BLOCKS© infused with glucose were created. On 12 June 2024, 24 L of bloom lake water was collected, and then 6 L was aliquoted into each of four 7 L mesocosms. Two HABS-BLOCKS© were added to each of two replicate treatment mesocosms, while two “Dummy” HABS-BLOCKS© (same but without glucose) were added to each of two control mesocosms. Cyanobacteria cell densities and microcystin concentrations were measured weekly. Total nitrogen and phosphorus and other water quality variables including dissolved oxygen, specific conductivity, and turbidity were measured at the end of the six-week experiment. Initially, *Microcystis* was measured at 2.4 × 10^3^ cells/mL and *Planktothrix* at 5.0 × 10^2^ cells/mL. After 6 weeks, both the *Microcystis* and *Planktothrix* population were significantly lower in the treated mesocosms compared to the controls. The initial microcystin concentration averaged 10.4 µg/L. By the third week of the experiment, the microcystin concentration was about 70% lower in the treated mesocosms. Total nitrogen was also lower in the HABS-BLOCKS©-treated mesocosm, but the other water quality measures were similar between the control and treated mesocosms. HABS-BLOCKS© appeared to reduce cyanobacterial cell densities, lower toxin concentrations, and lower total nitrogen while not having negative impacts on other water quality measures. Although much remains to be learned, this technology may someday be useful in suppressing HABS.

## 1. Introduction

Harmful cyanobacterial blooms (HCBs), also called harmful algal blooms (HABs), are increasing in frequency in lakes and reservoirs [1]. One of the most common cyanobacterial toxins in water bodies is microcystin. Microcystin is toxic to many organs in humans and animals [2] and has negative effects on most aquatic life [3,4]. Cyanobacterial toxins, like microcystin, threaten drinking water sources worldwide [5].

The World Health Organization recommends the short-term drinking water guidelines of 12 μg/L microcystin for adults and 3 μg/L for small children in finished water [6]. The United States Environmental Protection Agency supports more stringent drinking water guidelines of 1.6 and 0.3 μg/L, respectively [7].

Toxic chemicals like hydrogen peroxide or copper sulfate have been used to kill cyanobacteria and, as a result, mitigate HCBs/HABs [8]. However, once the bloom has formed, adding an oxidizer like hydrogen peroxide causes lysis of the cells and release of the toxins [9,10] and, even after treatment with hydrogen peroxide, the cyanobacterial populations return, requiring additional treatments [11]. Treatment of a bloom with copper sulfate was found to reduce the cyanobacterial population, but the surviving cyanobacterial genera demonstrated greater diversity and the potential for additional biological impacts [12]. Therefore, non-toxic treatment technologies are needed.

Previously, we showed that the addition of glucose to lake water mesocosms suppressed cyanobacteria and promoted the growth of heterotrophic bacteria [13,14]. The resulting hypothesis was generated that the glucose suppressed the cyanobacteria by promoting other, more competitive heterotrophic bacteria. We tested this hypothesis by treating lake water with glucose and, using the 16S rRNA sequence data, examined the bacterial community compositions in the treated and control mesocosms. Non-metric multidimensional scaling analysis showed that the resulting bacterial communities, i.e., the beta diversity, were significantly different in the treated and control mesocosms [15]. The heterotrophic bacteria, including Proteobacteria and Bacteroidota, grew to dominate the cyanobacteria in the glucose-treated mesocosms [15]. However, additional mechanisms may be involved in the suppression of the cyanobacteria resulting from adding glucose or HABS-BLOCKS© to lake water.

To target the glucose addition to the euphotic zone, floating, slow-release glucose agents designated HABS-BLOCKS© were created [16]. HABS-BLOCKS© are made of pumice stone infused with glucose and covered with soy wax. (However, other floating materials like wood blocks can also be used to carry the glucose and make HABS-BLOCKS©.) In this mesocosm study, our goal was to determine if HABS-BLOCKS© could suppress the cyanobacterial populations and lower microcystin concentrations in lake water collected from a drinking water reservoir experiencing a developing bloom.

## 2. Materials and Methods

HABS-BLOCKS© were produced, as previously described [16]. Briefly, pumice sticks (US Pumice Company, Chatsworth, CA, USA) were cut into 1 × 2 × 3 cm blocks, then infused with glucose under vacuum. The blocks were then placed in a drying oven at 40 °C to remove the excess water. After drying, the glucose-infused blocks, each containing about 3 g of glucose, were covered with soy wax (Northern Lights Natural Soy Wax, Wellsville, NY, USA) to make them a slow-release glucose product. The wax was melted, and then each block was covered with wax by dipping the block into the melted soy wax, which was then allowed to solidify. “Dummy” HABS-BLOCKS© are the same as HABS-BLOCKS© but without glucose. Previously, we have shown that HABS-BLOCKS©, under sterile conditions, released glucose rapidly the first week and then at gradually diminishing rates over the next 3 weeks [16].

In early June 2024, researchers noted a thick blue-green sheen across the surface of William H. Harsha Lake (hereafter, Lake) in Ohio, which is a man-made flood control reservoir that is also used as a source of drinking water [17]. These physical observations of the Lake water coincided with an increase in surface water pH and phycocyanin relative fluorescence units, which led the researchers to believe a harmful cyanobacteria bloom was occurring. On June 12, 2024, water was collected from the Lake’s surface (~0.5 m depth), using a 28 L cleaned and sterilized plastic water jug (Nalgene, Rochester, NY, USA). Once the water was returned to the laboratory and mixed, replicate 25 mL samples were diluted 100-fold for the enumeration and identification of cyanobacteria using the FlowCam Cyano trigger mode instrument, equipped with a 10× lens (Yokogawa Fluid Imaging Technologies, Scarborough, ME, USA) [18,19,20]. A combination of automated and manual sorting was used to classify the cyanobacteria into different groups in the Visual Spreadsheet version 5 software. Cyanobacteria were enumerated weekly in each mesocosm.

Next, 6 L of the Lake water was added to each of four 7 L polypropylene mesocosm vessels (8SFSPP, CAMBRO, Huntington Beach, CA, USA). Then, each mesocosm vessel was covered with a transparent xerography sheet (Skillcraft, Greensboro, NC, USA). The mesocosms were placed in an environmental chamber (Percival/166LLVL) with the following growth conditions: the light intensity was 44.02 µmol photons/m^2^/s (measured using a LICOR LI-1500) with a 16/8 h light/dark cycle at a constant temperature of 25 °C and ambient air exchange. After the 6 L of Lake water was added to each of the four mesocosms, the water was allowed to equilibrate for 24 h.

Next, two HABS-BLOCKS© or two “Dummy” HABS-BLOCKS© were added to replicate mesocosms to form the treated and control mesocosms, respectively. During the experiment, the mesocosms were mixed continuously using stir plates. The glucose concentration in each mesocosm was measured every 3 days using Glucose Test Strips (Precision Laboratories Inc., Cottonwood, AZ, USA) following the manufacturer’s instructions.

To measure microcystin concentrations in the water each week, replicate 5 mL aliquots were collected from each mesocosm and placed into 15 mL glass tubes (Pyrex, Corning, Corning, NY, USA). These samples were then frozen at −20 °C until they were analyzed for microcystin concentrations using ELISA, following EPA Method 546 [21] by BSA Environmental Sciences Inc. (Beachwood, OH, USA).

At the end of the experiment, 100 mL of water was collected from each mesocosm for total nitrogen and total phosphorus quantification using flow injection (Latchat Quickchem 8500, Hach Co, Loveland, CO, USA) with an alkaline persulfate digest followed by the cadmium reduction method for nitrate and an acidic persulfate digest followed by molybdenate colorimetry for orthophosphate, respectively [22]. Then, the YSI EXO2 (Yellow Springs Instruments, Yellow Springs, OH, USA) was used to measure water quality parameters, i.e., the percent saturation of dissolved oxygen, specific conductivity, total dissolved solids, and turbidity in each mesocosm.

## 3. Results

**Figure 1:**
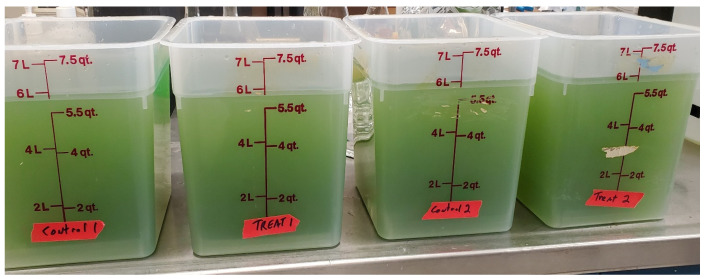
Appearance of mesocosms on 12 June 2024.

Figure 1 shows the appearance of the four mesocosms on June 12, 2024, before being placed in the incubator. FlowCam analysis of the Lake water showed that the initial *Planktothrix* concentration was 5.0 × 10^2^ cells/mL in the Lake water (Table 1). During the 6-week experiment, *Planktothrix* concentrations increased to an average of 3.7 × 10^4^ cells/mL in the control mesocosms but, in the HABS-BLOCKS©-treated mesocosms, the *Planktothrix* concentrations remained essentially unchanged (Table 1). Over the course of the six-week experiment, the average weekly *Planktothrix* concentration was significantly lower (*T*-test, *p* = 0.03) in the HABS-BLOCKS©-treated mesocosms compared to that in the control mesocosms.

The initial *Microcystis* concentration was 2.4 × 10^3^ cells/mL. The *Microcystis* concentration increased to an average 1.3 × 10^5^ cells/mL in the control mesocosms but only to an average of 1.3 × 10^4^ cells/mL in the HABS-BLOCKS©-treated mesocosms (Table 1). Over the course of the experiment, the average weekly *Microcystis* concentration in the HABS-BLOCKS©-treated mesocosms was significantly lower (*T*-test, *p* = 0.04) than that in the control mesocosms.

**Figure 2:**
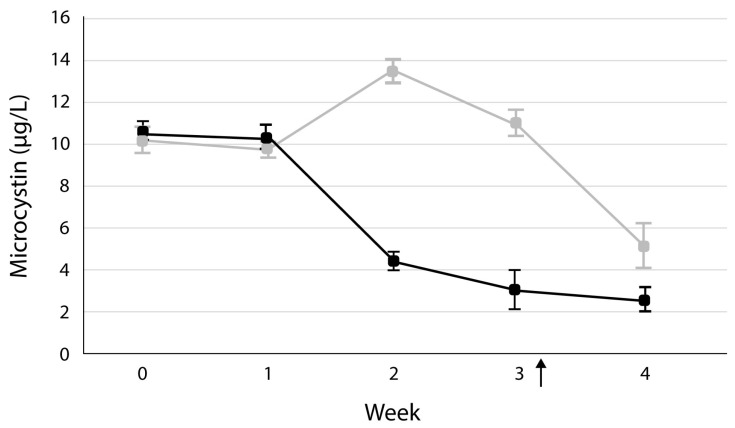
Average concentrations and standard deviations of microcystin in the treated (black dots) and control (gray dots) mesocosms for the first four weeks of the study. Two more HABS-BLOCKS© added at arrow.

The average microcystin concentration in the initial bloom water was 10.4 µg/L and was about the same after the first week (Figure 2). However, by the second week, the microcystin concentration had dropped to an average of 4.5 µg/L in the HABS-BLOCKS©-treated mesocosms, but the average microcystin concentration in the control mesocosms had increased to an average of 13.5 µg/L. By week 3, the microcystin concentration was 70% lower in the treated mesocosms compared to the control mesocosms (Figure 2).

**Figure 3:**
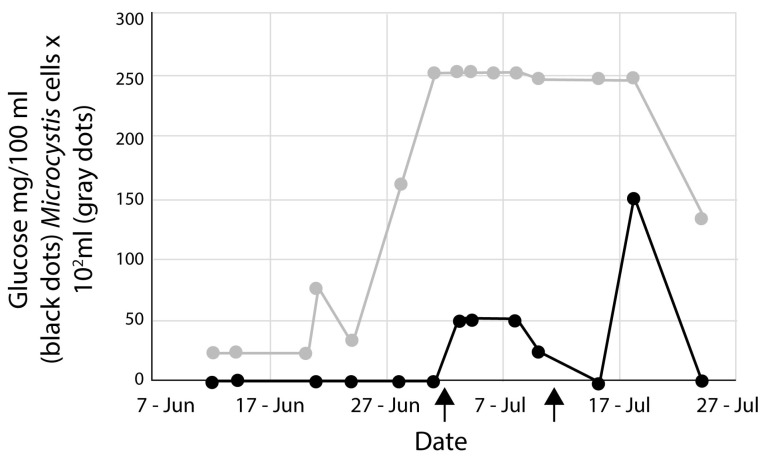
Average glucose concentrations (mg/100 mL) (black dots) and average *Microcystis* concentrations (×10^2^ cells/mL water) (gray dots) in mesocosms treated with two HABS-BLOCKS©. Two additional HABS-BLOCKS© were added on July 3 and July 15, as indicted at the arrows.

The glucose concentrations were tested in each mesocosm every three days during the experiment and compared to the average *Microcystis* concentrations in the HABS-BLOCKS©-treated mesocosm (Figure 3). Glucose was below the detection limit (25 mg/100 mL Lake water) during the first few weeks of the experiment, so on July 3, two additional HABS-BLOCKS© or “Dummy” HABS-BLOCKS© were added in each of the treated or control mesocosms, respectively. The concentration of glucose quickly reached about 50 mg/100 mL of water in the treated mesocosms but was again undetectable by July 15, at which time two additional HABS-BLOCKS© or “Dummy” HABS-BLOCKS© were added in each of the treated or control mesocosms, respectively. The glucose concentration increased in the treated mesocosms to an average of about 150 mg/100 mL, but the concentration dropped by the end of the experiment. Glucose was below the detection limit in the control mesocosms during the experiment.

The average Sonde readings in the HABS-BLOCKS©-treated and control mesocosms at the end of the experiment are shown in Table 2. There were no differences in water quality parameters in the treated and control mesocosms at the end of the experiment.

The average total nitrogen and phosphorous concentrations in the HABS-BLOCKS©-treated and control mesocosms at the end of the experiment are shown in Table 3. The total nitrogen was 42% lower (*T*-test, *p* < 0.001) in the HABS-BLOCKS©-treated mesocosms compared to the controls, but the total phosphorous concentrations did not differ significantly.

## 4. Discussion

The cyanobacterial bloom in Harsha Lake in early June 2024 contained *Microcystis* and *Planktothrix* populations. *Microcystis* is reportedly a very common cyanobacterial genus worldwide [23], and *Planktothrix* often forms toxic blooms in temperate freshwater lakes [24]. Since *Microcystis* and *Planktothrix* exhibit different optimum responses to light, their co-occurrence in bodies of water is common [25,26].

In our previous mesocosm studies [13,14,15,16], various concentrations of glucose were tested for controlling cyanobacterial growth. In 200 mL mesocosms, glucose was added at concentrations of 25 or 250 mg glucose/200 mL Harsha Lake water. Molecular analysis, including 16S rRNA gene sequencing and qPCR-based quantification of *Microcystis* target genes, showed that the glucose-treated mesocosm water contained significantly lower *Microcystis* and microcystin-producing mcy-gene copies than the control mesocosm water [13]. 

In 1000 L mesocosms filled with Muskegon Lake water, glucose at either 30 or 150 mg glucose/L caused a rapid proliferation of heterotrophic bacteria and a reduction in the relative abundance of cyanobacteria [14]. In a mesocosm experiment testing extended cyanobacterial control with glucose, 500 mL of Harsha Lake water was added to 7 L mesocosms. Then, 150 mg of glucose was added to the treated mesocosm, and the control mesocosm received no glucose. Each week, 500 mL of freshly collected Harsha Lake water was added to the control and treated mesocosms. The treated mesocosms also received an additional 150 mg of glucose each week. The treatment with glucose largely eliminated cyanobacterial taxa and increased the abundance of heterotrophic taxa during the 10-week experiment [15].

Because the weekly addition of glucose appeared to control the cyanobacteria for many weeks, we developed a slow-release glucose source called HABS-BLOCKS© [16]. These were designed to eliminate the need for repeated additions of glucose over time. In testing these HABS-BLOCKS©, we found that the treated mesocosms quickly became dominated by heterotrophic bacteria, and the Planktothrix and Cyanobium cyanobacteria were nearly eliminated [16].

In this current study, the addition of HABS-BLOCKS© to the Lake water inhibited the amplification of both *Microcystis* and *Planktothrix* populations. Both *Microcystis* and *Planktothrix* produce microcystins [27]. As a result of the decrease in their numbers in the treatment mesocosms, the microcystin concentrations were significantly (*p* = 0.05) lower in the HABS-BLOCKS©-treated mesocosms compared to the “Dummy” HABS-BLOCKS© control mesocosms. However, by week 4, the microcystin concentrations, even in the control mesocosms, began decreasing, indicating how difficult it is to maintain a bloom in the laboratory mesocosm.

Based on the earlier studies, we estimated that about 1 g of glucose, i.e., two HABS-BLOCKS© per six liters of Lake water, would be sufficient to suppress the cyanobacteria in this current study. Although adding two HABS-BLOCKS© to the treated mesocosms was apparently adequate to stop *Planktothrix* amplification, it was not adequate to stop *Microcystis*. Since glucose was not detected in the treated mesocosms for several weeks after the start of the experiment, more than two HABS-BLOCKS© appears to be needed to more adequately control *Microcystis*. Only after additional HABS-BLOCKS© were added was the glucose detected (Figure 3). Determining the optimum amount of glucose or number of HABS-BLOCKS© needed will require more testing.

To control cyanobacterial blooms, many physical processes, chemicals, and biological products have been tested. However, a review of the effectiveness of these treatments found them lacking and suggested that additional treatment technologies should be investigated to control cyanobacterial blooms [28]. Previously, we showed that the addition of glucose to lake water can promote the growth of heterotrophic bacteria [13,14,15]. In agreement with our findings, Li et al. [29] recently showed that the addition of dissolved organic carbon caused an imbalance of the carbon-to-phosphorous ratio that induced the competitive relationship between *Microcystis* and heterotrophic bacteria. The proliferation of heterotrophic bacteria led to a 98% inhibition of *Microcystis* growth [29].

Each year, lakes receive significant inputs of nutrients from wastewater treatment plants, surface runoff, and sediment [30]. Agricultural and other anthropogenic additions of nitrogen and phosphorus nutrients are reportedly responsible for the proliferation of cyanobacterial blooms worldwide [31,32]. Therefore, removing nutrients from lakes is a priority.

Adding HABS-BLOCKS© to the Lake water appears to promote denitrification with a 42% loss of total nitrogen by the end of the experiment in the HABS-BLOCKS©-treated mesocosms compared to the controls (Table 3). Since in most waters, denitrification is limited by the lack of available carbon [33,34], adding HABS-BLOCKS© appears to have provided the needed carbon for denitrification. Similar results were found when other carbon sources were used to promote denitrifying bacteria [35] and fungi [30]. Therefore, HABS-BLOCKS© may promote denitrifying microorganisms, as well as competitive heterotrophic bacteria.

In addition to nutrient inputs, lakes receive large quantities of organic carbon, primarily composed of large polymers and humic acids [29]. These large molecules must be metabolized by microorganisms to produce simple sugars, like glucose, before they can be utilized [36]. The time lag for the processing of these large molecules affects the interaction between heterotrophic bacteria and the cyanobacteria [37]. It appears that to directly affect the bloom development, a readily available carbon source, like glucose, needs to be in the right place, i.e., in the euphotic zone, at the right time, i.e., at the beginning of the bloom.

One potential problem with adding glucose in the form of HABS-BLOCKS© to a lake would be potential negative impacts on water quality and aquatic life. The water quality parameters evaluated in our study, including dissolved oxygen, showed no significant differences in the treated and control mesocosms. However, there is a concern that adding excessive amounts of glucose to a lake might result in the reduction of dissolved oxygen. Therefore, this parameter and other water quality measures will require careful monitoring.

This study was only designed as a proof-of-concept test, but the results suggest that the HABS-BLOCKS© application should be pursued further. There are many limitations to extrapolating from mesocosms studies to predict what would happen in a lake. Since the mesocosms were continuously mixed, the stratified distribution of *Microcystis* and *Planktothrix* was not possible. We also did not enumerate the cyanobacterial cells by microscopic counting. However, Pomati et al. [38] reported that automated monitoring with scanning flow cytometry is probably the most efficient method of monitoring the build-up of blooms. Lastly, the effect of adding HABS-BLOCKS© on the water quality conditions of a lake cannot be reproduced in a six-week mesocosm study. By week 4 of the experiment, the microcystin concentration was much lower even in the control mesocosms, indicating the difficulty of maintaining a healthy cyanobacterial bloom in the laboratory for an extended period. Ultimately, the dynamic conditions of a lake, including temperature changes, wind and mixing, and variations in light intensity, cannot be replicated in a mesocosm study.

## 5. Conclusions

Non-toxic treatment technologies are needed to reduce the impacts of cyanobacterial blooms. HABS-BLOCKS© might have an application for controlling cyanobacterial populations, but the use of HABS-BLOCKS© will need to be optimized for different conditions, and possible negative impacts must be carefully evaluated.

## Figures and Tables

**Table 1:** Average number of *Planktothrix* and *Microcystis* cells per mL water for each week of the experiment.

WEEK	0	1	2	3	4	5	6	*T*-Test
AVG Planktothrix × 10^2^ Treated	5	3	1	3	5	19	9	*p*-value
AVG Planktothrix × 10^2^ Control	5	8	18	115	232	497	373	
Planktothrix Treated vs. Control								0.03
AVG Microcystis × 10^2^ Treated	24	77	35	163	253	248	134	
AVG Microcystis × 10^2^ Control	24	156	234	389	426	749	1260	
Microcystis Treated vs. Control								0.04

**Table 2:** Mean Sonde readings in the treated and control mesocosms at the end of the six-week experiment.

	Control	Treated	*T*-Test
Test Measurements (Units)	Mean	Mean	*p*-Value
Total Dissolved Solids (mg/L)	147	166	0.35
Dissolved Oxygen (percent saturation)	88	79	0.20
Turbidity (formazin nephelometric units)	135	101	0.52
Conductivity (µS/CM)	225	253	0.38

**Table 3:** Mean concentration and standard deviation (SD) for the total nitrogen (N) and total phosphorus (P) in the HABS-BLOCKS©-treated and control mesocosms at the end of the experiment.

		Treated		Control		*T*-Test
Analyte	Unit	Mean	SD	Mean	SD	*p*-Value
Total Nitrogen	mg-N/L	4.930	0.801	8.37	0.338	<0.001
Total Phosphorus	mg P/L	0.545	0.011	0.405	0.177	0.17

## Data Availability

All data will be available at the NIH-PMC website.

## Abbreviations

The following abbreviations are used in this manuscript:

HCB	Harmful cyanobacteria bloom
HAB	Harmful algal bloom
P	Phosphorus
N	Nitrogen

## References

[1] Smucker N.J., Beaulieu J.J., Nietch C.T., Young J.L. (2021). Increasingly severe cyanobacterial blooms and deep-water hypoxia coincide with warming water temperatures in reservoirs. Glob. Change Biol..

[2] Ge K., Du X., Liu H., Meng R., Wu C., Zhang Z., Liang X., Yang J., Zhang H. (2024). The cytotoxicity of microcystin-LR: Ultrastructural and functional damage of cells. Arch. Toxicol..

[3] Wiegand C., Pflugmacher S. (2005). Ecotoxicological effects of selected cyanobacterial secondary metabolites: A short review. Toxicol. Appl. Pharmacol..

[4] Amorim C.A., Moura A.D.N. (2021). Ecological impacts of freshwater algal blooms on water quality, plankton biodiversity, structure, and ecosystem functioning. Sci. Total Environ..

[5] Lad A., Breidenbach J.D., Su R.C., Murray J., Kuang R., Mascarenhas A., Najjar J., Patel S., Hegde P., Youssef M. (2022). As we drink and breathe: Adverse health effects of microcystins and other harmful algal bloom toxins in the liver, gut, lungs and beyond. Life.

[6] World Health Organization (2020). Cyanobacterial toxins. Background Document for Development of WHO Guidelines for Drinking-Water Quality and Guidelines for Safe Recreational Water Environments.

[7] (2024). United States Environmental Protection Agency. https://www.epa.gov/habs/epa-drinking-water-health-advisories-cyanotoxins.

[8] Mehdizadeh Allaf M., Erratt K.J., Peerhossaini H. (2023). Comparative assessment of algaecide performance on freshwater phytoplankton: Understanding differential sensitivities to frame cyanobacteria management. Water Res..

[9] Chen C., Wang Y., Chen K., Shi X., Yang G. (2021). Using hydrogen peroxide to control cyanobacterial blooms: A mesocosm study focused on the effects of algal density in Lake Chaohu, China. Environ. Pollut..

[10] Kim W., Park Y., Jung J., Jeon C.O., Toyofuku M., Lee J., Park W. (2024). Biological and chemical approaches for controlling harmful microcystis blooms. J. Microbiol..

[11] Piel T., Sandrini G., Weenink E.F.J., Qin H., Herk M.J.V., Morales-Grooters M.L., Schuurmans J.M., Slot P.C., Wijn G., Arntz J. (2024). Shifts in phytoplankton and zooplankton communities in three cyanobacteria-dominated lakes after treatment with hydrogen peroxide. Harmful Algae.

[12] Watson S.E., Taylor C.H., Bell V., Bellamy T.R., Hooper A.S., Taylor H., Jouault M., Kille P., Perkins R.G. (2024). Impact of copper sulphate treatment on cyanobacterial blooms and subsequent water quality risks. J. Environ. Manag..

[13] Vesper S., Sienkiewicz N., Struewing I., Linz D., Lu J. (2022). Prophylactic addition of glucose suppresses cyanobacterial abundance in lake water. Life.

[14] Linz D., Partridge C.G., Hassett M.C., Sienkiewicz N., Tyrrell K., Henderson A., Tardani R., Lu J., Steinman A.D., Vesper S. (2024). Changes in cyanobacterial phytoplankton communities in lake-water mesocosms treated with either glucose or hydrogen peroxide. Microorganisms.

[15] Linz D., Struewing I., Sienkiewicz N., Steinman A.D., Partridge C.G., McIntosh K., Allen J., Lu J., Vesper S. (2024). Periodic addition of glucose suppressed cyanobacterial abundance in additive lake water samples during the entire bloom season. J. Water Resour. Prot..

[16] Vesper S., Linz D., Struewing I., Lu J. (2024). HABS-BLOCKS©, a floating, slow-release glucose source, promoted the growth of heterotrophic bacteria relative to toxic cyanobacteria in lake water mesocosms. J. Water Resour. Prot..

[17] Chen K., Allen J., Lu J. (2017). Community structures of phytoplankton with emphasis on toxic cyanobacteria in an Ohio inland lake during bloom season. J. Water Resour. Prot..

[18] Bertone E., Chuang A., Burford M.A., Hamilton D.P. (2019). In-situ fluorescence monitoring of cyanobacteria: Laboratory-based quantification of species-specific measurement accuracy. Harmful Algae.

[19] Mirasbekov Y., Zhumakhanova A., Zhantuyakova A., Sarkytbayev K., Malashenkov D.V., Baishulakova A., Dashkova V., Davidson T.A., Vorobjev I.A., Jeppesen E. (2021). Semi-automated classification of colonial *Microcystis* by FlowCAM imaging flow cytometry mesocosm experiment reveals high heterogeneity during seasonal bloom. Sci. Rep..

[20] Roache-Johnson K.H., Stephens N.R. (2023). FlowCam 8400 and FlowCam Cyano phytoplankton classification and viability staining by imaging flow cytometry. Methods Mol. Biol..

[21] (2016). United States Environmental Protection Agency. https://www.epa.gov/sites/default/files/2016-09/documents/method-546-determination-total-microcystins-nodularins-drinking-water-ambient-water-adda-enzyme-linked-immunosorbent-assay.pdf.

[22] Lipps W.C., Braun-Howland E.B., Baxter T.E., American Public Health Association, American Water Works Association, Water Environment Federation (2023). Standard Methods for the Examination of Water and Wastewater.

[23] Le Manach S., Duval C., Marie A., Djediat C., Catherine A., Edery M., Bernard C., Marie B. (2019). Global metabolomic characterizations of *Microcystis* spp. highlights clonal diversity in natural bloom-forming populations and expands metabolite structural diversity. Front. Microbiol..

[24] Pancrace C., Barny M.A., Ueoka R., Calteau A., Scalvenzi T., Pédron J., Barbe V., Piel J., Humbert J.F., Gugger M. (2017). Insights into the *Planktothrix* genus: Genomic and metabolic comparison of benthic and planktic strains. Sci. Rep..

[25] Torres Cde A., Lürling M., Marinho M.M. (2016). Assessment of the effects of light availability on growth and competition between strains of *Planktothrix agardhii* and *Microcystis aeruginosa*. Microb. Ecol..

[26] Trbojević I., Blagojević A., Kostić D., Marjanović P., Krizmanić J., Popović S., Simić G.S. (2019). Periphyton development during summer stratification in the presence of a metalimnetic bloom of *Planktothrix rubescens*. Limnologica.

[27] Gobler C.J., Burkholder J.M., Davis T.W., Harke M.J., Johengen T., Stow C.A., Van de Waal D.B. (2016). The dual role of nitrogen supply in controlling the growth and toxicity of cyanobacterial blooms. Harmful Algae.

[28] Anantapantula S.S., Wilson A.E. (2023). Most treatments to control freshwater algal blooms are not effective: Meta-analysis of field experiments. Water Res..

[29] Li T., Xu L., Li W., Wang C., Gin K.Y., Chai X., Wu B. (2024). Dissolved organic carbon spurs bacterial-algal competition and phosphorus-paucity adaptation: Boosting *Microcystis*’ phosphorus uptake capacity. Water Res..

[30] Liu T., Zhao Z., Li H., Awasthi M.K., Kosolapov D.B., Ni T., Ma B., Liu X., Liu X., Zhi W. (2024). Performance of aerobic denitrifying fungal community for promoting nitrogen reduction and its application in drinking water reservoirs. J. Environ. Manag..

[31] Brandenburg K., Siebers L., Keuskamp J., Jephcott T.G., Van de Waal D.B. (2020). Effects of nutrient limitation on the synthesis of N-rich phytoplankton toxins: A meta-analysis. Toxins.

[32] Wagner N.D., Quach E., Buscho S., Ricciardelli A., Kannan A., Naung S.W., Phillip G., Sheppard B., Ferguson L., Allen A. (2021). Nitrogen form, concentration, and micronutrient availability affect microcystin production in cyanobacterial blooms. Harmful Algae.

[33] Rajta A., Bhatia R., Setia H., Pathania P. (2020). Role of heterotrophic aerobic denitrifying bacteria in nitrate removal from wastewater. J. Appl. Microbiol..

[34] Yang S., Huang T., Zhang H., Tang Y., Guo H., Hu R., Cheng Y. (2024). Promoting aerobic denitrification in reservoir water with iron-activated carbon: Enhanced nitrogen and organics removal efficiency, and biological mechanisms. Environ. Res..

[35] Li Z., Huang T., Wu W., Xu X., Wu B., Zhuang J., Yang J., Shi H., Zhang Y., Wang B. (2024). Carbon slow-release and enhanced nitrogen removal performance of plant residue-based composite filler and ecological mechanisms in constructed wetland application. Bioresour. Technol..

[36] D’Souza G., Ebrahimi A., Stubbusch A., Daniels M., Keegstra J., Stocker R., Cordero O., Ackermann M. (2023). Cell aggregation is associated with enzyme secretion strategies in marine polysaccharide-degrading bacteria. Int. Soc. Microb. Ecol. J..

[37] Haraldsson M., Gerphagnon M., Bazin P., Colombet J., Tecchio S., Sime-Ngando T., Niquil N. (2018). Microbial parasites make cyanobacteria blooms less of a trophic dead end than commonly assumed. Int. Soc. Microb. Ecol. J..

[38] Pomati F., Jokela J., Simona M., Veronesi M., Ibelings B.W. (2011). An automated platform for phytoplankton ecology and aquatic ecosystem monitoring. Environ. Sci. Technol..

